# Molecular clue links bacteria to the origin of animals

**DOI:** 10.7554/eLife.00242

**Published:** 2012-10-15

**Authors:** Michael G Hadfield

**Affiliations:** **Michael G Hadfield** is in the Department of Biology and the Pacific Biosciences Research Center at the University of Hawaii at Manoa, Honolulu, United Stateshadfield@hawaii.edu

**Keywords:** Salpingoeca rosetta, Algoriphagus, bacterial sulfonolipid, multicellular development

## Abstract

Bacteria have a role in the formation of colonies by a species of single-celled organisms whose ancestors gave rise to the animals, which suggests that bacteria might also have influenced the origin of multicellularity in animals.

**Related research article** Alegado RA, Brown LW, Cao S, Dermenjian RK, Zuzow R, Fairclough SR, Clardy J, King N. 2012. A bacterial sulfonolipid triggers multicellular development in the closest living relatives of animals. *eLife* **1**:e00013. doi: 10.7554/eLife.00013

**Image** Colonies of the choanoflagellate *S. rosetta*

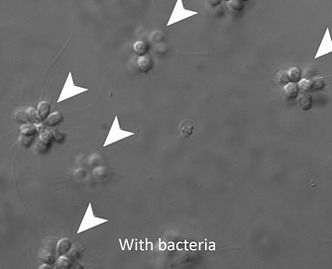

Over the last decade, phylogeneticists have reached a consensus about the branch of the tree of life that includes the fungi, the choanoflagellates and the animals (Parfrey et al., 2010). It is now clear that the choanoflagellates—aquatic organisms that contain just one or a few cells—and the animals are sister groups, which strongly suggests that animal multicellularity evolved within ancient choanoflagellates (Carr et al., 2008). Research by Nicole King of the University of California at Berkeley has shown that a single-celled species of choanoflagellate, *Salpingoeca rosetta*, can also exist in organized colonies, which are called rosettes (see Figure 1; King, 2004). Exploring the factors that cause the transition from single cells to colonies, King and co-workers have identified bacterial strains that serve as food for the choanoflagellates and induce the formation of colonies (Fairclough et al., 2010), and recently they described a new species of bacteria that is capable of inducing colony formation (Alegado et al., 2012b). Now, as reported in *eLife*, a collaboration led by King and Jon Clardy of Harvard Medical School has determined the identities of multiple bacterial species from a single phylum (the *Bacteroidetes*) that can induce these transitions. They have also established the chemical nature of molecules produced by the bacteria that are responsible for inducing colony formation in *S. rosetta* (Alegado et al., 2012a).

**Figure 1.**
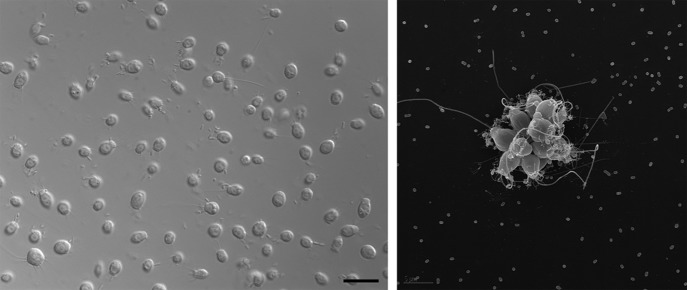
Electron micrographs showing single choanoflagellates (left; scale bar is 2 microns) and a colony of choanoflagellates (right; scale bar is 5 microns). The release of a sulfonolipid molecule by Algoriphagus bacteria that live among the choanoflagellates is thought to trigger the formation of the colonies.

FIGURE CREDIT: ROSIE ALEGADO (LEFT); MARK DAYEL (RIGHT).

While multicellularity must have arisen numerous times during evolution, this latest advance is notable because it exposes the role of bacteria in the process. Perhaps we should not be surprised, because bacteria had the earth to themselves for at least a billion years before single-celled eukaryotes evolved from and among them, and it is likely that bacteria were involved in all aspects of the lives of the early protists, including the beginnings of colony formation. However, it is surprising that bacteria have a role as central as that revealed in this work.

What is especially interesting about the bacteria-induced formation of colonies in *S. rosetta* is that it happens as a result of incomplete cell division, starting with a single cell, rather than the aggregation individual cells (Fairclough et al., 2010; Dayel et al. 2011). The process is thus reminiscent of animal embryology. Moreover, the cells in these choanoflagellate colonies reside within a common extracelluar matrix and are connected to each other by short (∼1 µm), complex intercellular bridges, much like those found between cells in many animal tissues (Dayel et al., 2011).

The collaboration, which includes Rosie Alegado of Berkeley and Laura Brown of Harvard as joint first authors, describes the complex process through which they isolated and identified the bacterial molecule that induces the transformation (Alegado et al., 2012a). The molecule is a sulfonolipid with the chemical formula C_32_H_64_NO_7_S. They speculate that this bacterial sulfonolipid has a highly specific receptor on the choanoflagellate surface, and that the binding of the sulfonolipid molecule to this receptor, which peaks at concentrations in the femtomolar range, may be related to feeding advantages enjoyed by the rosette-colony phase. The discovery of this distinct role for a sulfonolipid is certain to spur research on the occurrence and function of these compounds in many groups of organisms.

A major role for bacteria in colony formation in the closest living relatives of animals suggests another avenue of research, namely on the development of sponges. A major cell type in all sponges, the choanocyte, bears remarkable structural and functional similarity with the choanoflagellates, and this suggests a close evolutionary history between the two. The sponges, which are recognized as the simplest and probably most ancient of the animals, are hosts to many bacterial species: indeed, bacteria can make up more than 40% of the volume of an individual. Moreover, many bacterial species are transmitted, via embryos and larvae, from one generation of sponges to the next. There is also an intimate and complex relationship between cyanobacteria (bacteria that obtain their energy from photosynthesis) and the embryos of their sponge host. Furthermore, bacterial cells have also been observed in the intracellular vacuoles of developing sponges (see Thacker and Freeman, 2012 for a review of research into the interactions between sponges and microbes). All this work suggests a number of questions: do the interactions between bacteria and developing embryos in sponges have a role that is similar to the role played by bacteria in the formation of multicellular colonies in *S. rosetta*? Are symbiotic bacteria essential for normal sponge development?

The new insights into the interactions between bacteria and the earliest ancestors of the animals revealed by Alegado and co-authors, along with our growing understanding of the relationships between bacteria and sponges, should remind all biologists that bacteria are responsible for more than their impacts on our health—without them, our single-celled ancestors might never have made the transition to multicellularity. Like those early choanoflagellates, all living animals exist in a world filled with bacteria, and these bacteria are likely to be having an impact on many aspects of their biology.
